# Comparison of retrograde intrarenal surgery and standard percutaneous nephrolithotomy for management of stones at ureteropelvic junction with high-grade hydronephrosis

**DOI:** 10.1038/s41598-021-93551-8

**Published:** 2021-07-07

**Authors:** Fang Wang, Yun Hong, Zesong Yang, Liefu Ye

**Affiliations:** 1 Department of Clinical Medicine, Fujian Health College, Fuzhou, 350001 Fujian China; 2 Department of Urology, Fujian Provincial Hospital, Fuzhou, 350001 Fujian China; 3 Shengli Clinical Medical College, Fujian Medical University, Fuzhou, China

**Keywords:** Medical research, Urology

## Abstract

Percutaneous nephrostomy (PCNL) and retrograde intrarenal surgery (RIRS) are the two main treatments for upper urinary tract stones. The aim of our study was to compare the effectiveness and safety of standard PCNL (S-PCNL) and RIRS for the treatment of stones at ureteropelvic junction with high-grade hydronephrosis. The study included 118 patients who underwent surgery for stones at ureteropelvic junction. S-PCNL and RIRS were performed on 66 and 52 patients, respectively. Patient age, sex, body mass index (BMI), stone side, history of urinary tract infection (UTI), history of diabetes, history of ESWL, stone size, Hounsfield unit (HU) values of stones, grade of hydronephrosis, operating time, postoperative hemoglobin loss, narcotic analgesic use, postoperative transfusion rates, stone-free rates (SFRs), length of hospital stay, complication rates and number of secondary interventions were recorded. The comparison of the operative data between the two groups revealed no statistically significant differences in the operative time, SFRs, narcotic analgesic use, postoperative transfusion rate or other postoperative complications defined according to the Clavien system (P > 0.05). The postoperative urinary sepsis rate in the RIRS group was as high as 15.4%, which was much higher than the 1.5% rate observed in the S-PCNL group, and the difference was statistically significant (P < 0.05). A total of 13.5% of the patients in the RIRS group required a second operation due to failure of the placement of the ureteral access sheath. Additionally, S-PCNL had an advantage in operation time, while RIRS in duration of hospital stay and postoperative hemoglobin loss. RIRS and S-PCNL were safe and effective methods for the treatment of stones at ureteropelvic junction with high-grade hydronephrosis. Importantly, S-PCNL had more advantages in terms of the postoperative urinary sepsis rate and secondary surgery rate.

## Introduction

Urolithiasis is a common disease in urology that includes upper urinary tract stones and lower urinary tract stones. Among them, upper urinary tract stones account for the majority of cases. In recent years, the incidence of upper urinary tract stones has been increasing^1–3^. Larger stones often cause hydronephrosis, which eventually leads to the loss of kidney function on one side. Bilateral upper urinary tract obstruction can lead to acute renal failure. In addition, the pressure in the kidney increases after hydronephrosis, allowing bacteria to flow back into the blood circulation, which can lead to sepsis. These complications seriously threatens the health of patients.

Percutaneous nephrolithotomy (PCNL) and retrograde intrarenal surgery (RIRS) are the two main treatments for upper urinary tract stones. They have their own advantages and are widely used in the clinical setting. The main factors contributing to the choice of the treatment method for upper urinary tract stones include the location of stones, size of stones and diameter of the ureter. Stones at ureteropelvic junction are among the common types of upper urinary tract stones, often causing high-grade hydronephrosis^4^, and both PCNL and RIRS can effectively remove them. In general, PCNL provides significantly higher stone-free rates (SFRs) compared to RIRS at the expense of higher complication rates, such as bleeding, prolonged urine leaks and pleural injury^5^. Many doctors and patients often choose RIRS for safety reasons, especially in China. However, previous studies have not focused on this particular location of stones. Therefore, our aim in this study was to compare the efficacy and safety of PCNL and RIRS for the treatment of stones at ureteropelvic junction with high-grade hydronephrosis by retrospective analysis.

## Materials and methods

### Patients

A total of 1162 patients who underwent surgery for upper urinary tract stones in the Department of Urology of Fujian Provincial Hospital between June 2015 and December 2020 were retrospectively screened. Of these patients, 118 with stones at ureteropelvic junction and high-grade hydronephrosis were included in the study. These patients were divided into two groups according to the operation method. Standard PCNL (S-PCNL) and RIRS were performed on 66 and 52 of these 118 patients, respectively. For the treatment of patients, RIRS is mostly used in the early stage, and S-PCNL is mostly used in the later stage of that period. Patients with congenital kidney abnormalities, such as horseshoe kidneys and medullary sponge kidneys, and patients undergoing secondary operations due to residual stones were not included in the study.

Before the operation, complete blood counts, routine biochemistry, routine urine labs, urinary bacteria cultures, coagulation tests, electrocardiography, posterior-anterior chest radiography and kidney, ureter, and bladder (KUB) X-rays were performed for all the study patients. Computed tomography urography (CTU) images of the patients were acquired.

Patients with positive urine bacteria culture had been treated with sensitive antibiotics until the urine bacteria culture turned negative, and patients with diabetes had been treated with insulin until the fasting plasma glucose had been controlled below 8 mmol/L.

Noncontrast computed tomography (NCCT) images of the patients were performed on the seventh postoperative day as the first evaluation after surgery. The second evaluation of the patients was performed at the end of the fourth postoperative week through KUB X-ray and ultrasound examination. Double-J stents (F5) were placed in all patients during the operation and were removed under local anesthesia at the end of the fourth postoperative week. The patients who were not stone free underwent secondary procedures. All operations were performed by the same experienced surgeon.

Patient age, sex, body mass index (BMI), stone side, history of urinary tract infection (UTI), history of diabetes, history of extracorporeal shock wave lithotripsy (ESWL), stone size, Hounsfield unit (HU) values of stones, grade of hydronephrosis, operating time, postoperative hemoglobin loss (hemoglobin difference between the preoperative and the third postoperative days), narcotic analgesic use, postoperative transfusion rates, SFR, length of hospital stay, complication rates according to the modified Clavien system and number of secondary interventions were recorded.

### Hydronephrosis classification

Ipsilateral hydronephrosis was graded 0 to 4 according to CTU images: grade 0-no caliceal or pelvic dilatation, grade 1-pelvic dilatation only, grade 2-mild caliceal dilatation, grade 3-severe caliceal dilatation and grade 4-caliceal dilatation accompanied by renal parenchymal atrophy. Grade 3 and grade 4 hydronephrosis were defined as high-grade hydronephrosis^6–8^.

### Standard percutaneous nephrolithotomy

First, the patients were placed in the lithotomy position. A 5F ureteral catheter was placed into the ureter and accessed ureteropelvic junction on the operation side over a Cook 0.38 guidewire using cystoscopy. Then, the patients were turned into a prone position. Eighteen-gauge Chiba needles were used to achieve renal access under ultrasound guidance. A Cook 0.38 guidewire was placed into the renal pelvis on the side of surgery. Fascial dilators were used for dilation over the guidewire, and an F24 Amplatz sheath was placed in the kidney. Renal access was gained through the Amplatz sheath using a nephroscope (Wolf 8964.401 8F) device. A pneumatic and ultrasonic endoscopic lithotripter (Electro Medical Systems/EMS-IV) was used to dust and remove stones. An F20 nephrostomy tube was placed in each of the 66 patients.

### Retrograde intrarenal surgery

First, the patient was placed in the lithotomy position. Ureteral access was gained using a Storz 8F/9.8F rigid ureterorenoscope. A Cook 0.38 guidewire progressed to the renal pelvis on the side of surgery. A Cook 10–12F ureteral access sheath progressed over the guidewire. The kidney was accessed through the sheath using an electronic flexible ureteroscope (Storz Flex X2), and the stones were fragmented by a Ho-YAG (Lumenis PowerSuite 60w) device and a 220-lm laser fiber. Direct access was performed for two patients, and an F7 double-J stent was placed for seven patients in whom the intraoperative placement of the ureteral access sheath was not successful. These seven patients with double-J stents successfully underwent a secondary RIRS operation after 2 weeks.

### Statistical analysis

SPSS 20.0 software was used for the statistical analyses. The enumeration data are expressed as the mean ± standard deviation. The Wilcoxon test was used to test continuous variables that did not conform to a normal distribution. Student’s t-test was used to test continuous variables conforming to a normal distribution. The chi-square test was used for the comparison of the two study groups. A P value of < 0.05 was considered statistically significant.

### Research involving human participants

Informed consent was obtained from all subjects. The study was approved from by the Ethics Committee of Fujian Provincial Hospital. And we certify that the study was performed in accordance with the ethical standards as laid down in the 1964 Declaration of Helsinki and its later amendments or comparable ethical standards.

## Results

General information about the two groups of patients was summarized in Table 1. The comparison of the two study groups revealed no statistically significant differences in age, sex, BMI, stone side, stone size, HU values, grade of hydronephrosis, and history of ESWL, UTI and diabetes (P > 0.05).

**Table 1:** General information of the two groups.

Parameters	S-PCNL (n = 66)	RIRS (n = 52)	t/χ^2^ value	P value
Age (years)	51.33 ± 9.45	51.85 ± 9.23	−0.30	0.77
**Gender, n (%)**				
Male	27 (40.91)	23 (44.23)	0.13	0.72
Female	39 (59.09)	29 (55.77)		
BMI (kg/m^2^)	23.80 ± 2.08	23.64 ± 2.14	0.40	0.69
**Side of stones, n (%)**				
Right	30 (45.45)	26 (50.00)	0.24	0.62
Left	36 (54.55)	26 (50.00)		
Stone size (mm)	21.42 ± 3.60	20.19 ± 3.33	1.91	0.06
Stone CT density (HU)	1033.17 ± 282.53	1053.85 ± 245.05	−0.42	0.68
**Grade of hydronephrosis, n (%)**				
Grade 3	28 (42.42)	30 (57.69)	2.23	0.14
Grade 4	36 (54.55)	22 (42.31)		
History of ESWL, n (%)	2 (3.03)	3 (5.77)	–	0.65
History of UTI, n (%)	13 (19.70)	9 (17.31)	0.11	0.74
History of diabetes, n (%)	7 (10.61)	11 (21.15)	2.50	0.11

*BMI* body mass index, *CT* computed tomography, *HU* Hounsfield unit, *ESWL* extracorporeal shock-wave lithotripsy, *UTI* urinary tract infection, *S-PCNL* standard percutaneous nephrolithotomy, *RIRS* retrograde intrarenal surgery.

The comparison of the operative data between the two groups revealed no statistically significant differences in the operative time, SFR, narcotic analgesic use, postoperative transfusion rate or other postoperative complications defined according to the Clavien system (P > 0.05). The operation times were 51.83 ± 12.09 min and 54.42 ± 15.33 min in the S-PCNL and RIRS groups, respectively. The operation time was found to be shorter, with a statistically significant difference in the S-PCNL group compared with the RIRS group (P < 0.05). In contrast, in terms of the duration of hospital stay, the RIRS group was shorter than the S-PCNL group (P < 0.05). The postoperative hemoglobin loss values were 17.61 ± 8.55 g/L and 6.85 ± 4.76 g/L in the S-PCNL and RIRS groups, respectively, and there was a statistically significant difference between the two groups (P < 0.05). Although the S-PCNL group had higher postoperative hemoglobin loss values than the RIRS group, there were no patients requiring blood transfusion in either group. The postoperative urinary sepsis rate in the RIRS group was as high as 15.4%, which was much higher than the 1.5% in the S-PCNL group, and the difference was statistically significant (P < 0.05). Patients with postoperative urinary sepsis were cured by sensitive antibiotics. A total of 13.5% of the patients in the RIRS group required a second operation due to the failure of placement of the ureteral access sheath, while all the patients in the PCNL group completed the operation at one time (P < 0.05). Furthermore, the time of the secondary operation and the days of secondary hospitalization were included in the data. The operative data of the two groups are summarized in Table 2.

**Table 2:** Operation data.

Parameters	S-PCNL (n = 66)	RIRS (n = 52)	t/χ^2^ value	P value
Operating time (minutes)	51.83 ± 12.09	54.42 ± 15.33	−1.03	0.04
Stone-free rate, n (%)	65 (98.48)	49 (94.23)	–	0.32
Duration of hospital stay (days)	8.68 ± 1.39	5.77 ± 2.69	7.60	0.00
Postoperative hemoglobin loss (g/L)	17.61 ± 8.55	6.85 ± 4.76	8.13	0.00
Narcotic analgesic use, n (%)	3 (4.55)	1 (1.92)	–	0.63
Postoperative transfusion rate, n (%)	0 (0.00)	0 (0.00)	–	–
Postoperative urinary sepsis, n (%)	1 (1.52)	8 (15.38)	6.09	0.01
**Other postoperative complication, n (%)**				
Clavien 1	2 (3.03)	1(1.92)	–	> 0.99
Clavien 2	0 (0.00)	0 (0.00)	–	–
Clavien 3	1 (1.52)	0 (0.00)	–	> 0.99
Clavien 4–5	0 (0.00)	0 (0.00)	–	–
Secondary operation, n (%)	0 (0.00)	7 (13.46)	–	0.00

*S-PCNL* standard percutaneous nephrolithotomy, *RIRS* retrograde intrarenal surgery.

## Discussion

RIRS and PCNL are the most common operations in urology, which are known as minimally invasive procedures. The effectiveness and safety of these two operations for upper urinary tract stones have been confirmed by many studies^9,10^. However, due to the different approaches and advantages of the two operations, their clinical indications are also different.

In this study, both RIRS and S-PCNL showed high (> 90%) and similar SFRs, which confirmed that both procedures can effectively treat stones at the ureteropelvic junction. In terms of operation time, S-PCNL was shorter than RIRS. The reason may be that high-grade hydronephrosis makes the establishment of renal access easier and that ultrasonic endoscopic lithotripter is more efficient. However, the S-PCNL group showed a longer hospital stay.

It is worth noting that the postoperative urinary sepsis rate of RIRS was much higher than that of S-PCNL. There are many reasons for this result. First, high-grade hydronephrosis indicates that the upper urinary tract was completely blocked by the stone, which will cause a large number of bacteria to accumulate in the renal pelvis. Furthermore, RIRS has a smaller fluid outflow channel, resulting in higher intrarenal pelvic pressure during surgery. Doizi et al.^11^ have confirmed that the highest intrapelvic pressure of RIRS is 46.2 cm H_2_O in the kidney model, while the highest intrapelvic pressure of S-PCNL is only 7.3 cm H_2_O. In our clinic center, we also noticed that the incidence of infection after RIRS was relatively high, so PCNL was often used for surgery in the later stage. Since the intrapelvic pressure in mini-PCNL can be as high as 36.7 cm H_2_O^11^, we chose S-PCNL for treatment.

In our study, although S-PCNL had a higher postoperative hemoglobin loss than RIRS, the blood transfusion rate of the two groups was similar. Meanwhile, the rates of narcotic analgesic use and other postoperative complications of the two groups were also similar. There were no life-threatening complications in any of the two study groups.

In the RIRS group, 13.5% of patients failed to place the ureteral access sheath and chose to indwell the double-J stent for the secondary operation. In addition to the factor of narrowness of the ureter, this choice may be related to the distortion of the ureter under the stone in those patients. Repeated hospitalizations and surgeries increase the suffering of patients.

In addition, if the stone enters the kidney, high-grade hydronephrosis may cause difficulty in finding the stone and operating the flexible ureteroscope, which may affect the operation time and SFR. In contrast, for PCNL, high-grade hydronephrosis makes the establishment of percutaneous nephropathy easier, which may be the reason why the postoperative transfusion rate and incidence of other postoperative complication after PCNL such as pleural injury is not significantly higher compared to RIRS in this study.

There were various limitations of this study. Since our study was a retrospective design, the data may be biased. The number of patients included in the study was limited due to the inclusion of a single center. Further prospective studies on larger patient series are needed.

## Conclusion

RIRS and S-PCNL were safe and effective methods for the treatment of stones at ureteropelvic junction with high-grade hydronephrosis. Importantly, S-PCNL had more advantages in terms of the postoperative urinary sepsis rate and secondary surgery rate.
